# Oral health in people with schizophrenia: associations with behavioural and pharmacological factors

**DOI:** 10.1017/S2045796026100523

**Published:** 2026-03-26

**Authors:** Leire Urien, Ainara Arnaiz, Xabier Marichalar-Mendia, Unax Lertxundi, Jose de Leon, Agate Txurruka, Nerea Jauregizar, Teresa Morera-Herreras

**Affiliations:** 1 Department of Stomatology, Faculty of Medicine and Nursing, University of the Basque Country (UPV/EHU), Leioa, Spain; 2 Department of Pharmacology, Faculty of Medicine and Nursing, University of the Basque Country (UPV/EHU), Leioa, Spain; 3 Biobizkaia Health Research Institute, Mental Health Network, Barakaldo, Bizkaia, Spain; 4 Bizkaia Mental Health Network, Osakidetza Basque Health Service, Vizcaya, Spain; 5 Psychiatry Service, Galdakao-Usansolo University Hospital, Osakidetza Basque Health Service, Vizcaya, Spain; 6 Department of Nursing I, Faculty of Medicine and Nursing, University of the Basque Country (UPV/EHU), Leioa, Spain; 7 Bioaraba Health Research Institute, Osakidetza Basque Health Service, Araba Mental Health Network, Araba Psychiatric Hospital, Pharmacy Service, Vitoria-Gasteiz, Spain; 8 Basque Sustainable Pharmacy and Biotherapy Research Group, School of Pharmacy, University of the Basque Country (UPV/EHU), Vitoria-Gasteiz, Spain; 9 Mental Health Research Center, Eastern State Hospital, Lexington, KY, USA; 10 Biomedical Research Centre in Mental Health Net (CIBERSAM), Santiago Apóstol Hospital, University of the Basque Country, Vitoria, Spain; 11 Neurodegenerative Diseases Group, Biobizkaia Health Research Institute, Barakaldo, Bizkaia, Spain

**Keywords:** anticholinergic burden, DMFT index, oral health, periodontal disease, salivary flow, schizophrenia

## Abstract

**Aim:**

This study aimed to assess the oral health status of individuals with schizophrenia and explore its association with behavioural and pharmacological factors, with particular focus on long-term antipsychotic treatment and cumulative anticholinergic burden.

**Methods:**

A total of 153 adults with schizophrenia (18–65 years) from the Mental Health Network of Bizkaia (Spain), all under antipsychotic treatment for ≥12 months, were evaluated and compared with 153 controls from the general population. Data on socio-demographic variables, tobacco use and oral hygiene habits were collected. Oral health was assessed using the Decayed, Missing and Filled Teeth (DMFT) index and the Community Periodontal Index of Treatment Needs (CPITN). Unstimulated salivary flow was measured, and subjective xerostomia symptoms were recorded. Cumulative anticholinergic burden was estimated using the Drug Burden Index, considering both psychotropic and non-psychotropic medications. The association between dental health and clinical, behavioural and pharmacological variables was analysed in patients with schizophrenia.

**Results:**

Patients with schizophrenia exhibited significantly poorer oral health than controls, with higher mean DMFT scores (15.3 vs. 10.9; *p* < 0.001) and more advanced periodontal disease indicated by CPITN. Salivary hypofunction (<0.45 ml/min) was present in 31% of patients versus 12% of controls. In addition, high to very high anticholinergic burden was present in 71.9% of patients with schizophrenia, compared to only 3.3% of controls. In patients with schizophrenia, multivariate analyses identified the following as significant predictors of worse dental status (DMFT): age; smoking; female sex; illness duration; reduced salivary flow; poor tooth brushing; and anticholinergic burden. For periodontal health (CPITN), however, no variable was identified as a significant predictor of high-risk periodontal status.

**Conclusions:**

Oral health is substantially compromised in individuals with schizophrenia, reflecting a multifactorial interplay of behavioural, systemic and pharmacological factors. Both cumulative anticholinergic burden and reduced salivary flow independently contribute to poorer dental health, while periodontal disease appears to result from more complex influences not fully captured with studied variables. These findings underscore the importance of proactive clinical strategies, including regular dental assessments, targeted oral hygiene interventions, interdisciplinary collaboration between mental health and dental care providers and careful review of psychopharmacological regimens to minimize unnecessary anticholinergic exposure. Such integrated approaches are essential to preserve oral health, enhance quality of life and improve long-term outcomes in this vulnerable population.

## Introduction

Schizophrenia is a chronic and severe psychiatric disorder characterised by substantial functional impairment, increased physical morbidity and a significantly reduced life expectancy (Momen *et al.*, 2022; Chan *et al.*, 2023). Although the physical health of this population has attracted increased attention in recent years, oral health remains a relatively overlooked domain. A substantial body of evidence, including findings from our group, has demonstrated that individuals with schizophrenia experience a disproportionate burden of oral diseases, including dental caries, periodontal pathology, tooth loss and edentulism (Arnaiz *et al.*, 2011; Kisely *et al*., 2011; Chu *et al.*, 2012; Wey *et al.*, 2016; Denis *et al.*, 2019; Schoretsanitis *et al.*, 2020). Recent narrative reviews have further highlighted the persistence of oral health disparities in individuals with schizophrenia, particularly in ageing populations (Santhosh Kumar *et al.*, 2023).

This deterioration in oral health is widely recognised to be multifactorial. Contributing elements include poor oral hygiene, tobacco use, unhealthy dietary habits, cognitive impairment and limited access to dental services (Henderson *et al.*, 2006; Kebede *et al.*, 2019). Furthermore, negative symptoms and cognitive deficits can interfere with self-care and dental attendance (Arnaiz *et al.*, 2011). However, pharmacological factors – particularly long-term exposure to psychotropic medication – may also play a key role in oral health decline.

In this context, antipsychotic polypharmacy, defined as the concurrent use of two or more antipsychotics, is common in schizophrenia and is often associated with higher doses and prolonged treatment durations. The resulting increase in cumulative anticholinergic burden has received growing attention as a contributor to adverse health outcomes, particularly in people with complex and chronic conditions (Hsu *et al.*, 2021; Mehdizadeh *et al.*, 2021; Chengappa *et al.*, 2025). Anticholinergic effects, common to many psychotropic and systemic medications, have been associated with cognitive decline, cardiovascular dysfunction, frailty and general physical deterioration. One of the earliest and most noticeable manifestations is salivary hypofunction (Michail *et al.*, 2023), which compromises the oral environment by reducing the protective and homeostatic functions of saliva (Friedlander *et al.*, 2002; Llena-Puy, 2006). Reduced salivary flow can accelerate the development of dental caries and periodontal disease (Iorgulescu, 2009; Hu *et al.*, 2019).

In a recent pharmacovigilance study using the EudraVigilance database, we observed an elevated risk of oral adverse effects – particularly xerostomia – in patients treated with antipsychotics (Urien *et al.*, 2025), reinforcing the potential link between anticholinergic exposure and oral disease in this population. However, the relative contribution of anticholinergic burden to oral health, within the broader context of behavioural and clinical risk factors, remains insufficiently studied.

First, this study aims to assess oral health status in individuals with schizophrenia compared to matched controls. Second, in patients with schizophrenia, we aimed to examine dental and periodontal outcomes, salivary function and subjective xerostomia in relation to socio-demographic, behavioural and pharmacological factors, especially long-term antipsychotic use and cumulative anticholinergic burden.

## Methods

### Study design and participants

This descriptive, prospective, multicentre study was conducted over 5 years (January 2019–March 2024) to evaluate oral health outcomes in individuals with schizophrenia. The sample included 153 patients with a clinical diagnosis of schizophrenia, recruited from the administrative database of the Bizkaia Mental Health Network (Basque Country, Spain). Inclusion criteria were: (1) age 18–65 years; (2) diagnosis of schizophrenia according to the ICD-10 criteria (F20); and (3) continuous antipsychotic treatment for at least 12 months prior to enrolment. A control group of 153 individuals was recruited from the University of the Basque Country Dental Clinic and collaborating private dental practices. Controls had no history of schizophrenia and were not receiving antipsychotic medication. Exclusion criteria for both groups included: age <18 or >65 years, pregnancy, recent periodontal treatment (within 12 months), antibiotic use in the last 6 months, or current use of medications known to significantly affect oral health (e.g., antineoplastic agents, immunosuppressants, etc.). Individuals with prior head or neck radiotherapy were also excluded.

### Data collection

Sociodemographic data included age, sex, education and monthly income. Lifestyle and behavioural data covered tobacco and alcohol use, sugary drinks consumption, and use of cannabis or other illicit substances. Oral hygiene practices were self-reported (e.g., tooth brushing frequency, use of toothpaste, mouthwash, interdental aids). Additional information was collected on denture use, time since last dental visit and subjective symptoms such as dental pain, discomfort and dry mouth (xerostomia).

Comprehensive pharmacological data were extracted from clinical records, including all chronic prescriptions. On-demand and over-the-counter medications were excluded.

Anticholinergic burden was quantified using the Drug Burden Index (DBI), a validated measure estimating cumulative exposure to medications with anticholinergic and/or sedative properties via the formula DBI = *D*/*δ* + *D*, where *D* is the participant’s daily dose and *δ* the minimum effective daily dose sourced from national formularies and drug monographs (Hilmer *et al.*, 2007). DBI values were calculated using the Anticholinergic Burden Calculator (http://www.anticholinergicscales.es). All medications prescribed in the schizophrenia and control groups are detailed in Supplementary Tables S1 and S2, clearly distinguishing DBI-contributing drugs (with therapeutic class, DBI properties, *δ* values, patient exposure) from non-contributors.

Participants were classified into four DBI categories: low (DBI < 0.5), moderate (0.5–0.99), high (≥1) and very high burden (≥2). These thresholds are validated in older adult and psychiatric populations where DBI ≥ 1 predicts adverse outcomes (Martínez Arrechea *et al.*, 2021). The DBI dose-potency values (*δ* from formularies) follow the standard methodology validated across clinical populations, including psychiatric patients (Hilmer *et al.*, 2007; Chahine, 2023).

### Oral examinations

Oral health assessments were performed at each site by a trained dentist using portable instruments (explorer, intraoral mirror, WHO periodontal probe), following WHO diagnostic criteria.

#### Dental caries (DMFT index)

Dental status was evaluated using the Decayed, Missing and Filled Teeth (DMFT) index. The DMFT score ranges from 0 to 32 and is the sum of decayed (DT), missing (MT) and filled (FT) teeth. Higher scores indicate a greater cumulative burden of caries experience, reflecting both current pathology and prior treatment.

#### Periodontal health (CPITN index)

Periodontal status was assessed using the Community Periodontal Index of Treatment Needs (CPITN), based on clinical probing depth. The mouth was divided into sextants; the highest score per sextant was recorded and averaged. CPITN scores were classified as: (0) healthy; (1) gingival bleeding; (2) presence of supragingival or subgingival calculus; (3) periodontal pockets between 3.5 and 5.5 mm; and (4) periodontal pockets >5.5 mm.

#### Salivary flow rate

Unstimulated salivary flow was measured to assess salivary gland function. Participants were instructed to abstain from eating, drinking, smoking, or oral hygiene for at least one hour before testing. All samples were collected at the same time of day to control for circadian variation. Participants accumulated saliva for 60 seconds and expectorated into a pre-weighed Falcon tube. Flow rate was calculated, and classified as: hyposecretion (<0.45 ml/min), normal (0.45–1.35 ml/min), or hypersecretion (>1.35 ml/min).

### Statistical analysis

Data were analysed using IBM SPSS Statistics v29.0 (IBM Corp., Armonk, NY, USA). Continuous variables were expressed as means and standard deviations (SD), and categorical variables as frequencies and percentages. Group comparisons were performed using independent *t*-tests (continuous variables) and Chi-square (*χ*^2^) tests (categorical variables). In the group of patients with schizophrenia, the association between socio-demographic, behavioural and pharmacological factors and dental health (DMFT) was examined using univariate and multivariate linear regression models. For periodontal health (CPITN), the index was categorised as low risk (CPITN 1–2) or high risk (CPITN 3–4), and logistic regression analyses were conducted to identify potential predictors of high-risk periodontal status. All tests were two-tailed and statistical significance was set at *p*-value < 0.05.

### Ethical considerations

The study was conducted in accordance with the Declaration of Helsinki (1975, revised 2000). Approval was obtained from the Basque Country Clinical Research Ethics Committee (CEIm-Euskadi). All participants, or their legal guardians, provided written informed consent after receiving full details of the study. Personal identifiers were removed from the dataset, and all data were anonymised and managed in compliance with current data protection regulations.

## Results

### Demographic, medical and lifestyle characteristics of the study sample

The final sample included 153 patients with schizophrenia (104 males, 49 females; mean age 41.8 ± 13.7 years) and 153 controls (62 males, 91 females; mean age 44.4 ± 12.0 years). Detailed demographic, lifestyle and clinical information is presented in Table 1. Most patients with schizophrenia had been diagnosed for over 15 years, reflecting a chronically affected and long-term treated population.

**Table 1.** Demographic characteristics and anamnestic data

	Control group (*n*=153)	Schizophrenia group (*n*=153)
**Age (years)**		
≤30	44 (28.8)	7 (4.6)
31–41	29 (19.0)	35 (22.9)
42–52	40 (26.1)	68 (44.4)
≥53	40 (26.1)	43 (28.1)
**Gender**		
Female	91 (59.5)	49 (32.0)
Male	62 (40.5)	104 (68.0)
**Socio-economic status**		
Level of education^#^		
No education/primary education	27 (17.6)	80 (52.3)
Secondary/university education	126 (82.4)	71 (46.4)
Monthly income^#^		
Low income (<600€)	21 (13.7)	85 (55.6)
High income (>600€)	132 (86.3)	62 (40.5)
**Toxic habits**		
Non-smokers	107 (69.9)	46 (30.1)*
Tobacco (cigarettes/day)	1.1 (5.8)	13.7 (12.2)*
Weekly alcoholic beverages	0.5 (1.2)	13.7 (12.8)*
Marijuana	0.2 (1.3)	1.4 (7.0)*
Other drugs of abuse (psychostimulants)	0 (0)	19 (12.4)
Daily sugary drinks^#^		
<3/day	147 (96.1)	132 (86.3)
3–6/day	6 (3.9)	10 (6.5)
>6/day	0 (0)	9 (5.9)
**Oral hygiene habits**		
Oral care products^#^		
One product	98 (64.0)	120 (78.4)
>1 product	52 (34.0)	19 (12.4)
No product	3 (2.0)	14 (9.2)
Visit to the dentist in the last year	121 (79.1)	81 (52.9)
Visit to the dentist more than 5 years ago	7 (4.6)	23 (15.0)*
**Oral feelings**		
Sensation of oral discomfort	25 (16.3)	32 (20.9)
Subjective dry mouth sensation	18 (11.8)	48 (31.4)*
Dental pain	37 (24.2)	19 (12.4)
**Medical condition**		
Systemic disease	39 (25.5)	69 (45.1)*
Duration of mental illness		
5 years	–	12 (7.9)
5–15 years	–	36 (23.5)
>15 years	–	105 (68.6)

*Note*: Data are expressed as *n* (%) or mean (SD).

* *p* < 0.05, Student’s *t*-test.

# *p* < 0.05, Chi-square test.

Significant group differences emerged in lifestyle behaviours and general health indicators. Tobacco use was substantially higher among schizophrenia patients: only 46 were non-smokers, compared to 107 in the control group (*p* < 0.001). Cannabis or other illicit drug use (e.g., amphetamines, methamphetamines and other psychostimulants) was reported by 12.4% of patients, versus 0.2% of controls.

Oral hygiene habits also differed: while nearly all participants reported toothpaste use, only 14.4% of schizophrenia patients used more than one hygiene product (e.g., floss, mouthwash), versus 34.0% of controls. Dental attendance was lower in the schizophrenia group: 52.9% had visited a dentist in the past year (vs. 79.1% of controls), and 15% had not had a dental visit in over 5 years (vs. 4.6% of controls). Xerostomia was reported by 31.4% of patients, significantly more than in controls (11.8%).

Regarding pharmacological treatment, 88.9% of patients were treated exclusively with atypical antipsychotics and 11.1% with both typical and atypical antipsychotics. For analysis, patients were grouped into ‘atypical-only’ and ‘mixed-treatment’. Monotherapy was observed in 45.1% (*n* = 69), while 54.9% received antipsychotic polypharmacy (44.4% with two agents (*n* = 68), 9.8% with three (*n* = 15) and one patient with four (0.7%)). A total of 57 distinct pharmacological profiles were identified, reflecting the high heterogeneity of treatment regimens in this cohort. Clozapine use was notably high (47.7%, *n* = 73), far exceeding regional data from 2017 (16.2%), suggesting either a clinical shift or selection bias related to illness severity (Sanz-Fuentenebro *et al.*, 2019). The most frequent combinations included clozapine with aripiprazole (6.5%, *n* = 10) and clozapine with paliperidone (5.2%, *n* = 8) (see Table S1 for full distribution).

Concomitant psychotropic and non-psychotropic medication was common in the schizophrenia group (detailed in Table S2). Lithium was prescribed in 8.5% of cases (*n* = 13), while antidepressants and benzodiazepines were used by 17.6% and 47.0% of patients, respectively, compared to 1.3% and 5.2% in the control group. In addition to these agents, many schizophrenia patients were also receiving other medications with well-established anticholinergic effects, several of which are included in recognised reference lists of drugs contributing to anticholinergic burden (Byrne *et al.*, 2018).

### Anticholinergic burden, salivary flow and dry mouth symptoms

Given the high rates of psychotropic polypharmacy in the schizophrenia group, we assessed salivary function and cumulative anticholinergic burden, both of which are known to influence oral health. Although no significant difference in mean salivary flow was observed between groups (0.4 ± 0.4 ml/min in schizophrenia vs. 0.8 ± 0.9 ml/min in controls; *p* > 0.05), subgroup analyses revealed notable sex differences. Women with schizophrenia exhibited significantly lower salivary flow than women in the control group (0.6 ± 0.5 vs. 0.8 ± 0.4 ml/min; *p* < 0.001), whereas no such difference was found among men.

To account for inter-individual variability, salivary flow was categorised into three levels: low (<0.45 ml/min), normal (0.45–1.35 ml/min) and high (>1.35 ml/min). Compared to controls, schizophrenia patients showed a significantly higher frequency of both hyposalivation and hypersalivation (*p* < 0.05). Sex-stratified analysis indicated that low flow was more frequent among women (42.9%) and excessive flow among men (21.2%) in the schizophrenia group (*p* < 0.05). Subjective reports of xerostomia mirrored these findings: 31.4% of patients with schizophrenia reported dry mouth, compared to only 11.8% of controls (*p* < 0.001). While the type of antipsychotic treatment (atypical vs. mixed-treatment) was not associated with xerostomia prevalence, patients using antidepressants or benzodiazepines had a higher frequency of this symptom, suggesting additive effects of polypharmacy.

Anticholinergic burden, calculated using the DBI, was markedly higher among schizophrenia patients (1.7 ± 0.7 vs. 0.2 ± 0.4; *p* < 0.001). Nearly all patients (95.2%) fell within moderate to very high-risk categories: 23.3% with moderate burden (DBI 0.5–0.99), 39.0% with high burden (≥1) and 32.9% with very high burden (≥2). In contrast, only 9.8% of controls were classified in these categories, with 6.5% showing moderate and 3.3% high burden, while none presented very high burden. These findings reflect the frequent use of medications with known anticholinergic properties in this population, extending beyond antipsychotic treatment alone. Interestingly, anticholinergic burden was negatively correlated with salivary flow (*r* = −0.179, *p* < 0.05).

Given the crucial role of saliva in maintaining oral health and the established link between anticholinergic burden and salivary dysfunction, these results suggest a potential cumulative impact on oral health status. The following section details the dental condition of participants and its association with these and other clinical and behavioural factors.

### Dental status assessment: DMFT index

Dental health, assessed by the DMFT index, differed significantly between schizophrenia patients and controls (Table 2). The mean DMFT score was 15.3 ± 8.4 in patients versus 10.9 ± 7.2 in controls (*p* < 0.001). Patients had more decayed (1.7 ± 2.1 vs. 0.9 ± 1.2; *p* < 0.001) and missing teeth (8.9 ± 9.1 vs. 5.5 ± 6.7; *p* < 0.001), while filled teeth numbers were similar. Complete edentulism was also more common in the schizophrenia group (*n* = 11 vs. *n* = 2).

**Table 2.** Dental status parameters for control and schizophrenia groups

	Control group					Schizophrenia group				
	*n*	DT	MT	FT	DMFT	*n*	DT	MT	FT	DMFT
All participants	153	0.9 (1.2)	5.5 (6.7)	4.4 (4.0)	10.9 (7.1)	153	1.7 (2.1)*	8.9 (9.2)*	4.1 (4.0)	15.3 (8.4)*
**Gender**										
Females	91	0.6 (1.1)	5.1 (7.3)	4.5 (4.0)	10.3 (7.5)	49	1.4 (1.6)*	7.8 (8.4)*	4.8 (4.2)	14.6 (7.9)*
Males	62	1.3 (2.3)	6.1 (5.8)	4.2 (4.0)	11.7 (6.3)	104	1.7 (2.3)	9.3 (9.5)*	3.8 (3.9)	15.6 (8.5)*
**Age**										
<30	44	0.4 (1.4)	1.2 (1.7)	2.4 (3.0)	4.5 (4.2)	7	2.6 (4.3)*	2.4 (1.9)	2.7 (2.8)	7.9 (5.5)
31–41	29	1.7 (3.1)	3 (2.4)	5 (4)	9.7 (5.3)	35	2.3 (2.1)	3.0 (3.1)	4.5 (3.9)	9.3 (5.9)
42–52	40	0.8 (1.2)	5.9 (5.9)	6.5 (4.1)	13.2 (4.9)	68	1.6 (2.1)*	8.3 (7.8)*	4.6 (3.6)*	14.9 (6.9)
≥53	40	0.6 (1.0)	11.7 (8.3)	4.2 (3.6)	16.5 (6.7)	43	1.0 (1.4)	15.4 (10.8)*	4.1 (4.7)	21.9 (7.4)*
**Smoking**										
Non-smokers	107	0.7 (1.3)	4.9 (6.2)	4.7 (4.1)	10.5 (6.8)	46	1.3 (7.3)*	6.8 (6.4)*	4.6 (4.3)	13.3 (7.3)*
Smokers (>5 cigarettes/day)	33	1.6 (2.7)	7.9 (8.4)	3.7 (3.6)	13.2 (7.9)	102	1.9 (2.3)	10.0 (10.2)	3.8 (3.8)	16.4 (8.6)*
**Salivary flow**										
<0.45 ml/min	18	0.3(0.7)	6.9 (8.9)	4.1 (4.0)	11.3 (8.4)	48	1.4 (2.0)*	12.1 (11.3)	4.1 (4.7)	18.7 (9.3)*
0.45–1.35 ml/min	119	1 (1.9)	5.1(6.3)	4.2 (4.0)	10.4 (6.9)	82	1.9 (2.3)*	7.7 (8.0)*	4.0 (3.6)	14.1 (7.8) *
>1.35 ml/min	16	1.1 (1.4)	7.1 (7.0)	5.7 (3.9)	13.9 (6.3)	23	1.3 (1.5)	6.17 (6.0)	4.2 (3.9)	12.5 (6.7)
**Antipsychotic treatment**										
Atypical antipsychotics	**–**	**–**	**–**	**–**	**–**	136	1,7 (2,2)	8,4 (8,8)	4,2 (4,4)	14,9 (8,1)
Mixed-treatment	**–**	**–**	**–**	**–**	**–**	17	1,2 (1,5)	13,1 (11,6)^#^	3,6 (4,1)	19,5 (9,3)^#^

*Note*: Data are expressed as mean (SD). DT: decayed teeth; MT: missing teeth; FT: filled teeth; DMFT: decayed-missing-filled teeth score.

* *p* < 0.05 vs. control group, Student’s *t*-test;

# *p* < 0.05 vs. atypical antipsychotics, Student’s *t*-test.

Stratified by sex, both men and women with schizophrenia had higher DMFT scores and tooth loss than controls, though more decayed teeth were seen only in women. Age analyses showed younger (<30) and middle-aged (42–52) patients had more decayed teeth, while tooth loss increased with age (42–52 and ≥53). Those ≥53 years had the highest DMFT scores, reflecting cumulative damage.

DMFT remained significantly higher in schizophrenia patients regardless of smoking status. On the other hand, patients on combined typical and atypical antipsychotics showed greater tooth loss and DMFT scores than those on atypicals alone, suggesting either greater pharmacological burden or greater illness severity.

Reduced salivary flow correlated inversely with DMFT scores, highlighting the protective role of saliva. Anticholinergic burden was positively associated with DMFT, indicating that cumulative exposure to such medications contributes to dental deterioration. Lifestyle factors such as tobacco use were associated with increased dental damage, while marijuana use and frequent brushing were linked to lower DMFT scores (Table 3).

**Table 3.** DMFT risk-factor analysis in patients with schizophrenia

	Univariate analysis		Multivariate analysis (adjusted)	
	*R*	*p*	*β*	*p*
Gender (female)	0.148	**<0.01**	0.101	**<0.05**
Age	0.602	**<0.001**	0.583	**<0.001**
Illness duration	0.408	**<0.001**	0.352	**<0.01**
Monthly income	−0.113	0.05	−0.102	0.231
Smoking	0.271	**<0.001**	0.184	**<0.001**
Alcohol	0.040	0.485	−0.068	0.424
Marijuana	−0.134	**<0.05**	−0.948	0.345
Sugary drinks	0.028	0.632	0.102	0.230
Tooth brushing	−0.297	**<0.001**	−0.254	**<0.01**
Visits to the dentist	0.022	0.697	0.003	0.975
Salivary flow	−0.159	**<0.01**	−0.120	**<0.01**
Anticholinergic burden	0.197	**<0.05**	0.108	**<0.001**

*Note: β*: beta coefficient from multivariate analysis; *r*: Pearson’s correlation coefficient. Multivariate analysis was adjusted by age and gender.

Multivariate analyses identified age, smoking, female sex, illness duration, reduced salivary flow, poor tooth brushing and anticholinergic burden as significant predictors of poor dental status (Table 3).

### Periodontal status assessment: CPITN index

Assessment of CPITN revealed a significantly poorer status in schizophrenia patients compared to controls (*p* < 0.001; Table 4). The mean CPITN index in the schizophrenia group was 2.2 ± 1.0, significantly higher than the 1.5 ± 1.6 observed in the control group.

**Table 4.** Periodontal status parameters for control and schizophrenia groups

	Control group						Schizophrenia group						
	*n*	CPITN = 0	CPITN = 1	CPITN = 2	CPITN = 3	CPITN = 4	*n*	CPITN = 0	CPITN = 1	CPITN = 2	CPITN = 3	CPITN = 4	*p* (*χ*^2^)
All participants	153	40 (30.7)	20 (13.1)	56 (36.6)	27 (17.6)	3 (2)	153	14 (9.2)	13 (8.5)	59 (38.6)	56 (36.6)	11 (7.2)	<0.001
**Gender**													
Females	91	33 (36.3)	9 (9.9)	33 (36.3)	15 (16.5)	1 (1.1)	49	6 (12.2)	5 (10.2)	15 (30.6)	21 (42.9)	2 (4.1)	<0.05
Males	62	14 (22.6)	11 (17.7)	23 (37.1)	12 (19.4)	2 (3.2)	104	8 (7.8)	8 (7.8)	44 (42.3)	35 (34.3)	9 (8.7)	<0.05
**Age**													
<30	44	22 (50.0)	4 (9.1)	16 (36.4)	2 (4.5)	0 (0)	7	0 (0)	1 (14.3)	3 (42.9)	3 (42.9)	0 (0)	<0.05
31–41	29	9 (31.0)	3 (10.3)	13 (44.8)	4 (13.8)	1 (3.4)	35	2 (5.7)	6 (17.1)	14 (40.0)	13 (37.1)	0 (0)	<0.05
42–52	40	14 (35.0)	6 (15.0)	12 (30.0)	7 (17.5)	1 (2.5)	68	2 (3.0)	5 (7.3)	27 (39.7)	27 (39.7)	8 (11.8)	<0.001
≥53	40	2 (5.0)	7 (17.5)	16 (40.0)	14 (35.0)	1 (2.5)	43	10 (23.3)	1 (2.3)	15 (34.9)	15 (34.9)	2 (4.7)	NS
**Smoking**													
Non-smokers	107	38 (35.5)	15 (14.0)	35 (32.7)	17 (15.9)	2 (1.9)	46	1 (2.2)	3 (6.5)	23 (50.0)	18 (39.1)	1 (2.2)	<0.001
Smokers (>5 cigarettes/day)	33	6 (18.2)	3 (9.1)	15 (45.5)	8 (24.2)	1 (3.0)	102	12 (12.0)	9 (9.0)	35 (35.0)	37 (37.0)	9 (9.0)	NS
**Salivary flow**													
<0.45 ml/min	18	5 (27.8)	2 (11.1)	7 (38.9)	3 (16.7)	1 (5.6)	48	8 (16.7)	4 (8.3)	14 (29.2)	17 (35.4)	5 (10.4)	NS
0.45–1.35 ml/min	119	39 (32.8)	13 (10.9)	45 (37.8)	20 (16.8)	2 (1.7)	82	5 (6.1)	9 (11.0)	38 (46.3)	26 (31.7)	4 (4.9)	<0.001
>1.35 ml/min	16	3 (18.8)	5 (31.3)	4 (25.0)	4 (25.0)	0 (0)	23	1 (4.3)	0 (0)	7 (30.7)	13 (56.5)	2 (8.7)	<0.05
**Antipsychotic treatment**													
Atypical antipsychotics	**–**	**–**	**–**	**–**	**–**	**–**	136	11 (8.1)	13 (9.6)	49 (36.0)	54 (39.7)	9 (6.6)	NS
Mixed-treatment	**–**	**–**	**–**	**–**	**–**	**–**	17	3 (17.6)	0 (0)	10 (58.8)	2 (11.8)	2 (11.8)	

*Note*: Data are expressed as *n* (%). CPITN = 0: healthy, no evidence of periodontal disease; CPITN = 1: gingival bleeding; CPITN = 2: supragingival or subgingival calculus; CPITN = 3: pathological pockets of 3.5–5.5 mm; CPITN = 4: pathological pockets >5.5 mm. *p* (*χ*^2^): *p*-value; Chi-square test.

Although the CPITN = 2 category was the most common in both groups, the distribution of scores was markedly different. In the control group, the majority of individuals (80.4%, *n* = 123) fell into the healthier categories (CPITN = 0, 1 or 2). Conversely, 82.4% (*n* = 126) of patients with schizophrenia were clustered in the higher risk categories (CPITN = 3 or 4), indicating more severe periodontal conditions. Importantly, the most severe level (CPITN = 4), representing deep periodontal pockets and the need for complex treatment, was almost two and a half times more common in the schizophrenia group (7.2%) than in the controls (3%).

The difference in periodontal health between groups persisted after stratification by sex and age, with patients with schizophrenia consistently having worse scores than controls – except in the subgroup aged ≥53 years, where periodontal conditions were comparable. This convergence at older ages may reflect a general age-related deterioration in periodontal health that masks group differences. Interestingly, when only non-smokers were analysed, people with schizophrenia continued to have significantly worse periodontal status, suggesting that factors beyond tobacco use contribute to the oral health gap. In contrast, among smokers, no significant differences in CPITN scores were observed between groups, possibly indicating a ceiling effect of smoking-related periodontal damage. Unlike findings related to dental status, pharmacological treatment did not appear to influence periodontal health outcomes.

A logistic regression analysis was conducted to identify potential risk factors for periodontal disease in patients with schizophrenia, categorising CPITN scores into two levels: low risk (CPITN = 1–2) and high risk (CPITN = 3–4). However, none of the variables included in the model were statistically significant in predicting high periodontal risk (see Table 5). These results imply that periodontal disease in individuals with schizophrenia is probably caused by a complex interplay of behavioural, biological and pharmacological factors, and the measured variables did not fully capture.

**Table 5.** CPITN risk-factor analysis in patients with schizophrenia

	Logistic regression			
		95%CI		
	OR	Lower	Upper	*p*
Gender (female)	1	**–**	**–**	**–**
Gender (male)	0.829	0.419	1.641	0.590
Age	1.001	0.971	1.031	0.961
Illness duration	0.995	0.964	1.028	0.777
Monthly income (<600€)	1	**–**	**–**	**–**
Monthly income (>600€)	0.983	0.507	1.908	0.960
Smoking	1.016	0.990	1.042	0.226
Marijuana	1.010	0.963	1.058	0.689
Sugary drinks	0.939	0.799	1.102	0.439
Tooth brushing	0.749	0.539	1.046	0.090
Visits to the dentist (in the last 6 months)	1	**–**	**–**	**–**
Visits to the dentist (in the last year)	1.042	0.414	2.624	0.930
Visits to the dentist (in the last 2 years)	4.042	0.558	3.907	0.433
Visits to the dentist (in the last 5 years)	0.671	0.203	2.213	0.512
Visits to the dentist (more than 5 years)	2.768	0.997	7.686	0.051
Salivary flow	1.348	0.864	2.103	0.189
Anticholinergic burden	0.850	0.551	1.311	0.461

*Note*: OR: odds ratio; CI: confidence interval. The CPITN index was categorised into two levels: *low risk* (CPITN = 1–2) and *high risk* (CPITN = 3–4).

## Discussion

This study confirms that individuals with schizophrenia present significantly poorer oral health than the general population, both in prevalence and severity. This deterioration appears to result from a combination of behavioural, clinical and pharmacological factors. In particular, cumulative exposure to anticholinergic medications – frequent in this population – was independently associated with higher DMFT scores. While this relationship may be partly explained by reduced salivary flow and disrupted oral homeostasis, the independent contribution of both variables suggests that anticholinergic burden may influence dental health through additional mechanisms beyond xerostomia.

### Poor oral health in Schizophrenia

Our findings confirm that poor oral health is markedly more frequent in people with schizophrenia, with a DMFT index approximately 1.5 times higher than in controls. This difference was driven primarily by a greater number of decayed and missing teeth, whereas no significant differences were found in filled teeth. This pattern suggests both a higher burden of untreated dental disease and persistent barriers to restorative dental care, a problem consistently described in this population (Arnaiz *et al.*, 2011; Chu *et al.*, 2012; Wey *et al.*, 2016; Velasco-Ortega *et al.*, 2017; Aghasizadeh Sherbaf *et al.*, 2024). The absence of differences in fillings contrasts with Bertaud-Gounot *et al.* (2013), who observed fewer restorations in schizophrenia patients, suggesting potential variability in service availability or health policies across settings.

Edentulism was present in 7.2% of patients, a prevalence consistent with prior reports ranging from 4% to 14% (Chu *et al.*, 2012; Bertaud-Gounot *et al.*, 2013; Wey *et al.*, 2016; Velasco-Ortega *et al.*, 2017; Schoretsanitis *et al.*, 2020), and aligns with data on increased residual roots (Hernández-Suastegui and Vivanco-Cedeño, 2004), which may act as chronic inflammatory foci. In a 2011 systematic review, Kisely *et al*. reported edentulism prevalences as high as 65% in older studies of severe mental illness. Data from state psychiatric hospitals in the early 2000s showed rates around 20% (Schoretsanitis *et al.*, 2020), likely reflecting earlier treatment eras dominated by first-generation antipsychotics (de Leon *et al.*, 2005). Compared with these historical data, the prevalence observed in our sample (7.2%) may indicate a gradual improvement in dental care access and pharmacological management, although continued vigilance is warranted.

Both men and women with schizophrenia showed significantly worse dental status than their respective controls, although no sex differences were observed within the patient group. This contrasts with earlier studies suggesting poorer dental outcomes among men (Chu *et al.*, 2012; Denis *et al.*, 2019), possibly due to earlier illness onset and lower adherence to oral hygiene. DMFT scores increased with age, reflecting cumulative exposure to illness-related, behavioural and pharmacological risk factors (Velasco *et al.*, 1997; Bertaud-Gounot *et al.*, 2013; Wey *et al.*, 2016; Denis *et al.*, 2018). Indeed, studies reporting higher DMFT scores often included older samples (Wey *et al.*, 2016).

Tobacco use, markedly more prevalent in the schizophrenia group, was positively associated with dental damage, confirming its role as a major risk factor (Arnaiz *et al.*, 2011; Tani *et al.*, 2012). However, poorer dental health persisted even among non-smokers, supporting the contribution of additional determinants.

Pharmacological treatment, particularly cumulative anticholinergic burden, emerged as a key factor. Higher DBI scores were independently associated with worse DMFT values, even after adjusting for confounders. Patients receiving a combination of typical and atypical antipsychotics showed more missing teeth and higher DMFT values, supporting the hypothesis that polypharmacy and prolonged anticholinergic exposure aggravate dental deterioration (Grinshpoon *et al.*, 2015). Benzodiazepines and antidepressants, both widely used in our sample, also contribute to xerostomia and salivary gland dysfunction (Cockburn *et al.*, 2017), which diminish the natural protective role of saliva against caries (Wey *et al.*, 2016; Ngo *et al.*, 2018). Our findings extend previous research by highlighting that anticholinergic burden in schizophrenia, widely recognised for its cognitive implications (Chengappa *et al.*, 2025), also has significant relevance for oral health. As shown in our study and previous work (Chapuis *et al.*, 2020), cumulative anticholinergic exposure markedly contributes to dental disease by impairing salivary function and disrupting oral microbial balance.

### Periodontal vulnerability in schizophrenia

Our findings confirm markedly poorer periodontal health in individuals with schizophrenia compared to controls. Only 9.2% of patients showed a healthy periodontal status versus 30.7% of controls, while severe disease (CPITN =  3–4) was over twice as prevalent (43.8% vs. 20.6%). These results align with previous reports of elevated periodontal burden in schizophrenia (Kenkre *et al.*, 2000; Eltas *et al.*, 2013).

Periodontal impairment was observed across all age and sex groups, with severe disease (CPITN = 4) slightly more frequent in men (8.7% vs. 4.1%). Logistic regression using dichotomised CPITN scores (low risk = 1–2; high risk = 3–4) found no variable significantly predicting high periodontal risk. This lack of associations suggests that the elevated periodontal burden in schizophrenia reflects its multifactorial aetiology, involving biological susceptibility, medication effects and lifestyle factors beyond the model’s scope. Longitudinal studies are needed to assess the cumulative effects of illness, treatment and oral hygiene habits over time.

Beyond identifying risk factors, attention must be directed toward improving care delivery. Structured oral health promotion programs tailored to people with severe mental illness, including supervised toothbrushing interventions, motivational strategies and integration of oral screening within psychiatric services, have shown promise in reducing oral morbidity (Kou *et al.*, 2020; Joury *et al.*, 2025). Multidisciplinary collaboration between dental and mental health professionals is essential to reduce the persistent oral health gap observed in schizophrenia (Santhosh Kumar *et al.*, 2023).

Further studies should include larger samples and try to quantify the long-term effects of the variables. Future research would also benefit from balanced sex distributions or stratified analyses, given the observed differences in sex composition between patient and control groups and the known influence of sex on both schizophrenia and oral health outcomes. Ideally, longitudinal designs would better clarify causal pathways, although maintaining follow-up in this population remains challenging. Improved methodological designs will help disentangle the relative contribution of behavioural, systemic and pharmacological determinants and guide effective preventive strategies.

## Conclusions

This study underscores that oral health in people with schizophrenia remains a major yet often overlooked clinical issue. The findings highlight the multifactorial nature of poor oral health in this population, where behavioural, systemic and pharmacological factors, particularly the anticholinergic burden, contribute to caries and tooth loss, while periodontal disease appears to result from a more complex interplay of influences not fully captured in the variables that we studied.

Clinically, the findings call for proactive prevention through regular dental follow-up, targeted hygiene interventions and coordinated care between mental health and dental professionals. Implementation of structured oral health promotion programs, integration of routine dental screening within psychiatric services and education of caregivers and mental health staff in basic oral assessment may further help reduce long-term dental morbidity. Integrating oral health promotion into psychiatric services and reviewing psychopharmacological regimens to limit unnecessary anticholinergic exposure may help reduce dental morbidity and improve overall quality of life.

## Supporting information

10.1017/S2045796026100523.sm001 Urien et al. supplementary material Urien et al. supplementary material

## Data Availability

The data and code used for the analysis are not publicly available due to ethical concerns. However, upon reasonable request to the corresponding author, access to the data and code will be provided.

## Supplementary material

The supplementary material for this article can be found at https://doi.org/10.1017/S2045796026100523.
