# Supplementary material for: Oral health in people with schizophrenia: associations with behavioural and pharmacological factors
Source: Epidemiol Psychiatr Sci. 2026 Mar 26;35:e17. doi: 10.1017/S2045796026100523 (PMC13122550; doi:10.1017/S2045796026100523)
Supplement: Urien et al. supplementary material [file S2045796026100523sup001.docx]

**SUPPLEMENTARY MATERIAL**

**Oral health in people with schizophrenia: associations with behavioural and pharmacological factors**

Leire Urien^1,2^, Ainara Arnaiz^3^, Xabier Marichalar-Mendia^4^, Unax Lertxundi^5^, Jose de Leon^6,7^, Agate Txurruka^2^, Nerea Jauregizar^2,3,^ *^LA^*, Teresa Morera-Herreras^2,8,^ *^LA*^*

^1^Department of Stomatology, Faculty of Medicine and Nursing, University of the Basque Country (UPV/EHU), 48940-Leioa, Spain.

^2^Department of Pharmacology, Faculty of Medicine and Nursing, University of the Basque Country (UPV/EHU), 48940-Leioa, Spain.

^3^Grupo Red de Salud Mental, Biobizkaia Bizkaia Health Research Institute, Barakaldo, Bizkaia, Spain.

^4^Department of Nursing I, University of the Basque Country (UPV/EHU), Barrio Sarriena s/n, 48940, Leioa, Bizkaia, Spain.

^5^Bioaraba Health Research Institute; Osakidetza Basque Health Service, Araba Mental Health Network, Araba Psychiatric Hospital, Pharmacy Service, Vitoria-Gasteiz. Spain.

^6^Mental Health Research Center, Eastern State Hospital, Lexington, KY, USA

^7^Biomedical Research Centre in Mental Health Net (CIBERSAM), Santiago Apóstol Hospital, University of the Basque Country, Vitoria, Spain

^8^Neurodegenerative diseases Group, Biobizkaia Health Research Institute, Barakaldo, Bizkaia, Spain.

*^LA^* N Jauregizar and T Morera-Herreras should be considered shared last authors

**CONTENTS**

**Supplementary Tables (2):**

**Table S1.** Distribution of antipsychotic treatment profiles in the schizophrenia group

**Table S2.** Medications in the schizophrenia and control groups

**Table S1. Distribution of antipsychotic treatment profiles in the schizophrenia group**

| **TREATMENT PROFILE** | **n** | **%** |
| --- | --- | --- |
| Clozapine | 28 | 18.3 |
| Paliperidone | 18 | 11.8 |
| Aripiprazole + Clozapine | 10 | 6.5 |
| Clozapine + Paliperidone | 8 | 5.2 |
| Aripiprazole | 7 | 4.6 |
| Clozapine + Olanzapine | 6 | 3.9 |
| Olanzapine | 5 | 3.3 |
| Zuclopentixol | 4 | 2.6 |
| Clozapine + Risperidone | 4 | 2.6 |
| Aripiprazole + Olanzapine | 4 | 2.6 |
| Risperidone | 3 | 2.0 |
| Clozapine + Quetiapine | 3 | 2.0 |
| Cariprazine + Paliperidone | 2 | 1.3 |
| Amisulpride + Quetiapine | 2 | 1.3 |
| Cariprazine | 2 | 1.3 |
| Olanzapine + Risperidone | 2 | 1.3 |
| Clozapine + Zuclopentixol | 2 | 1.3 |
| Aripiprazole + Quetiapine | 2 | 1.3 |
| Aripiprazole + Clozapine + Lurasidone | 2 | 1.3 |
| Haloperidol + Paliperidone | 2 | 1.3 |
| Aripiprazole + Risperidone | 2 | 1.3 |
| Clotiapine + Olanzapine + Quetiapine | 1 | 0.7 |
| Asenapine + Haloperidol | 1 | 0.7 |
| Quetiapine + Zuclopentixol | 1 | 0.7 |
| Haloperidol + Olanzapine | 1 | 0.7 |
| Clozapine + Haloperidol + Quetiapine | 1 | 0.7 |
| Haloperidol + Quetiapine | 1 | 0.7 |
| Clotiapine + Olanzapine | 1 | 0.7 |
| Aripiprazole + Clotiapine | 1 | 0.7 |
| Clozapine + Haloperidol | 1 | 0.7 |
| Clozapine + Quetiapine + Risperidone | 1 | 0.7 |
| Clozapine + Levosulpiride + Paliperidone | 1 | 0.7 |
| Haloperidol | 1 | 0.7 |
| Haloperidol + Risperidone | 1 | 0.7 |
| Aripiprazole + Clotiapine + Clozapine | 1 | 0.7 |
| Olanzapine + Quetiapine | 1 | 0.7 |
| Clotiapine + Quetiapine | 1 | 0.7 |
| Lurasidone | 1 | 0.7 |
| Amisulpride + Clozapine + Quetiapine | 1 | 0.7 |
| Cariprazine + Clozapine + Olanzapine + Quetiapine | 1 | 0.7 |
| Amisulpride + Olanzapine | 1 | 0.7 |
| Aripiprazole + Olanzapine + Quetiapine | 1 | 0.7 |
| Aripiprazole + Quetiapine + Zuclopentixol | 1 | 0.7 |
| Clotiapine + Clozapine + Olanzapine | 1 | 0.7 |
| Paliperidone + Risperidone | 1 | 0.7 |
| Aripiprazole + Lurasidone | 1 | 0.7 |
| Lurasidone + Olanzapine | 1 | 0.7 |
| Aripiprazole + Paliperidone | 1 | 0.7 |
| Olanzapine + Paliperidone | 1 | 0.7 |
| Clozapine + Fluphenazine | 1 | 0.7 |
| Aripiprazole + Clozapine + Paliperidone | 1 | 0.7 |
| Fluphenazine + Olanzapine | 1 | 0.7 |
| Olanzapine + Paliperidone + Risperidone | 1 | 0.7 |
| Paliperidone + Quetiapine | 1 | 0.7 |
| Clozapine + Levomepromazine + Risperidone | 1 | 0.7 |
| Aripiprazole + Asenapine + Clozapine | 1 | 0.7 |
| **TOTAL** | **153** | **100** |

**Table S2.** **Medications in the schizophrenia and control groups.** Drugs without anticholinergic/sedative burden (Drug Burden Index (DBI) non-contributors) are shown in italics and marked with an asterisk.

| **DRUG** | **WHO ATC CODE** | **ANTICHOLINERGIC (AC) / SEDATIVE (S) EFFECTS** | **MINIMUN EFFECTIVE DAILY ORAL DOSE (MG)** | **SCHIZOPHRENIA (n)** | **CONTROL (n)** |
| --- | --- | --- | --- | --- | --- |
| **Antipsychotics** | | | | | |
| Amisulpride | N05AL05 | S | 50 | 4 | - |
| Aripiprazole | N05AX12 | S | 10 | 35 | - |
| Asenapine | N05AH05 | S | 10 *(sublingual)* | 2 | - |
| Cariprazine | N05AX15 | S | 1.5 | 5 | - |
| Clotiapine | N05AH06 | AC/S | 120 | 6 | - |
| Clozapine | N05AH02 | AC/S | 25 | 73 | - |
| Fluphenazine | N05AF02 | AC/S | 0.36 | 2 | - |
| Haloperidol | N05AD01 | S | 0.5 | 9 | - |
| Levomepromazine | N05AA02 | AC/S | 37.5 | 1 | - |
| Levosulpiride | N05AB02 | S | 50 | 1 | - |
| Lurasidone | N05AE05 | S | 37 | 5 | - |
| Olanzapine | N05AH03 | AC/S | 5 | 29 | - |
| Paliperidone | N05AX13 | S | 3 | 37 | - |
| Quetiapine | N05AH04 | AC/S | 50 | 19 | - |
| Risperidone | N05AX08 | S | 1 | 16 | - |
| Zuclopenthixol | N05AF05 | AC/S | 20 | 8 | - |
| **Benzodiazepines/hypnotics** | | | | | |
| Lorazepam | N05BA06 | S | 0.5 | 37 | 3 |
| Diazepam | N05BA01 | S | 1 | 13 | 1 |
| Clonazepam | N03AE01 | S | 0.5 | 11 | - |
| *Chlorazepate** | N05BA05 | S | - | 5 | - |
| Alprazolam | N05BA12 | S | 0.5 | 4 | - |
| Flurazepam | N05CD01 | S | 15 | 1 | - |
| Flunitrazepam | N05CD03 | S | 0.5 | 1 | - |
| Zolpidem | N05CF02 | S | 5 | - | 4 |
| **Antidepressants** | | | | | |
| **SSRI** | | | | | |
| Sertraline | N06AB06 | S | 50 | 4 | - |
| Fluoxetine | N06AB03 | S | 20 | 4 | - |
| Escitalopram | N06AB10 | S | 5 | 3 | - |
| Paroxetine | N06AB05 | AC/S | 20 | 1 | 1 |
| **SNRI** | | | | | |
| Duloxetine | N06AX21 | S | 30 | 3 | - |
| Venlafaxine | N06AX16 | S | 75 | 2 | - |
| **Tricyclic** | | | | | |
| Clomipramine | N06AA04 | AC/S | 30 | 2 | - |
| Amitriptyline | N06AA09 | AC/S | 10 | 1 | - |
| Nortriptyline | N06AA10 | AC/S | 30 | 1 | - |
| **Others** | | | | | |
| Trazodone | N06AX05 | S | 100 | 3 | - |
| Mirtazapine | N06AX11 | S | 15 | 2 | 1 |
| *Bupropion** | N06AX12 | - | - | 1 | - |
| **Mood stabilizers/impulse control** | | | | | |
| *Lithium** | N05AN01 | - | - | 13 | - |
| Valproic acid | N03AG01 | S | 1000 | 1 | - |
| Topiramate | N03AX11 | S | 50 | 1 | - |
| **Analgesics** | | | | | |
| Pregabalin | N03AX16 | S | 150 | 2 | 4 |
| Gabapentin | N03AX12 | S | 900 | 1 | 5 |
| Tramadol | N02AX02 | S | 200 | - | 2 |
| *Celecoxib** | M01AH01 | - | - | - | 1 |
| **Anticholinergic-Spasmolytic** | | | | | |
| Biperiden | N04AA02 | AC/S | 1 | 14 | - |
| Atropine | S01FA01 | AC | 0.6 | 1 | - |
| Solifenacin | G04BD08 | AC | 5 | 1 | - |
| Fesoterodine | G04BD11 | AC | 4 | 1 | - |
| Flavoxate | G04BD02 | AC/S | 600 | - | 1 |
| **Antihypertensives** | | | | | |
| Doxazosin | C02CA04 | S | 2 | 1 | - |
| *Propranolol** | C07AA05 | - | - | 1 | 3 |
| *Furosemide** | C03CA01 | - | - | 1 | - |
| **Antidiabetics** | | | | | |
| *Metformin** | A10BA02 | - | - | 10 | - |
| **Corticosteroids** | | | | | |
| *Methylprednisolone** | H02AB04 | - | - | 1 | - |
| *Fluticasone-Salmeterol** | R03AK06 | - | - | 1 | - |
| **Other drugs** | | | | | |
| *Methotrexate** | L04AX03 | - | - | - | 1 |
| Meclozine | R06AE05 | AC/S | 25 | - | 1 |

* Drugs not included in the list of medications used in the calculation of the DBI (Byrne et al. 2018; 8(7):e022500)

WHO ATC: World Health Organization Anatomical Therapeutic Classification; n = patients exposed
